# Identification of Gene Signature as Diagnostic and Prognostic Blood Biomarker for Early Hepatocellular Carcinoma Using Integrated Cross-Species Transcriptomic and Network Analyses

**DOI:** 10.3389/fgene.2021.710049

**Published:** 2021-09-29

**Authors:** Olfat Al-Harazi, Ibrahim H. Kaya, Maha Al-Eid, Lina Alfantoukh, Ali Saeed Al Zahrani, Mohammed Al Sebayel, Namik Kaya, Dilek Colak

**Affiliations:** ^1^ Department of Biostatistics, Epidemiology, and Scientific Computing, King Faisal Specialist Hospital and Research Centre, Riyadh, Saudi Arabia; ^2^ AlFaisal University, College of Medicine, Riyadh, Saudi Arabia; ^3^ Gulf Centre for Cancer Control and Prevention, King Faisal Special Hospital and Research Centre, Riyadh, Saudi Arabia; ^4^ Liver and Small Bowel Transplantation and Hepatobiliary-Pancreatic Surgery Department, King Faisal Specialist Hospital and Research Centre, Riyadh, Saudi Arabia; ^5^ Department of Surgery, University of Almaarefa, Riyadh, Saudi Arabia; ^6^ Translational Genomics Department, Center for Genomic Medicine, King Faisal Specialist Hospital and Research Centre, Riyadh, Saudi Arabia

**Keywords:** hepatocellular carcinoma, diagnosis, prognosis, gene signature, blood, transcriptome, early HCC, gene network

## Abstract

**Background:** Hepatocellular carcinoma (HCC) is considered the most common type of liver cancer and the fourth leading cause of cancer-related deaths in the world. Since the disease is usually diagnosed at advanced stages, it has poor prognosis. Therefore, reliable biomarkers are urgently needed for early diagnosis and prognostic assessment.

**Methods:** We used genome-wide gene expression profiling datasets from human and rat early HCC (eHCC) samples to perform integrated genomic and network-based analyses, and discovered gene markers that are expressed in blood and conserved in both species. We then used independent gene expression profiling datasets for peripheral blood mononuclear cells (PBMCs) for eHCC patients and from The Cancer Genome Atlas (TCGA) database to estimate the diagnostic and prognostic performance of the identified gene signature. Furthermore, we performed functional enrichment, interaction networks and pathway analyses.

**Results:** We identified 41 significant genes that are expressed in blood and conserved across species in eHCC. We used comprehensive clinical data from over 600 patients with HCC to verify the diagnostic and prognostic value of 41-gene-signature. We developed a prognostic model and a risk score using the 41-geneset that showed that a high prognostic index is linked to a worse disease outcome. Furthermore, our 41-gene signature predicted disease outcome independently of other clinical factors in multivariate regression analysis. Our data reveals a number of cancer-related pathways and hub genes, including *EIF4E, H2AFX, CREB1, GSK3B, TGFBR1,* and *CCNA2*, that may be essential for eHCC progression and confirm our gene signature’s ability to detect the disease in its early stages in patients’ biological fluids instead of invasive procedures and its prognostic potential.

**Conclusion:** Our findings indicate that integrated cross-species genomic and network analysis may provide reliable markers that are associated with eHCC that may lead to better diagnosis, prognosis, and treatment options.

## Introduction

Hepatocellular carcinoma (HCC) is a leading cause of cancer-related deaths worldwide (Bray et al., 2018; Yang et al., 2019a; Villanueva, 2019). The disease is mostly diagnosed at advanced stages, and therefore has poor prognosis. Liver biopsy is an invasive technique with potential difficulties, including risk of death, and is susceptible to sampling errors (Shi et al., 2014). HCC is usually diagnosed using serum alpha-fetoprotein (*AFP*) and ultrasound (Schütte et al., 2014; Tsuchiya et al., 2015) (Shi et al., 2014). Since 1964, the *AFP* biomarker has been most commonly used biomarker for HCC evaluation (Schütte et al., 2014; Tsuchiya et al., 2015). However, *AFP* has low sensitivity and specificity; therefore, the measurement of serum *AFP* levels has been discarded from updated international surveillance guidelines (Schütte et al., 2014). Hence, there is a significant need to identify alternative or additional biomarkers that can be used in effectively diagnosing patients in an early tumor stage.

The use of high-throughput technologies together with bioinformatics approaches in cancer research gives the ability to identify molecules implicated in complex pathways in carcinogenesis. Several recent studies have used whole-genome gene expression profiling, DNA methylation profiles, copy number variations (CNVs) in HCC in order to achieve a better understanding of the processes of hepatocarcinogenesis (Colak et al., 2010; Budhu et al., 2013; Shi et al., 2014; Allain et al., 2016; Wang et al., 2017; Guan et al., 2020; Li et al., 2020). It has been reported that combining genomic and network-based analysis could lead to reliable and accurate predictive biomarkers for human diseases (Colak et al., 2013; Al-Harazi et al., 2016). The molecular characteristics of HCC are heterogeneous which results in dissimilarities in the outcome of affected patients. Detecting the early stage tumors is essential as liver resection, transplantation or local ablation are the most effective treatment option for patients at early disease stages. However, HCC is usually diagnosed at advanced stages, and therefore, has poor prognosis (Waghray et al., 2015; Llovet et al., 2016). Some of the key factors that have an effect on patient survival include number and size of nodules, vascular invasion, existence of extrahepatic metastases, and liver function (Schütte et al., 2014). However, the survival of patients with the identified tumor stages still remains heterogeneous and some patients have early recurrence of disease after treatment or liver transplantation. Therefore, it is essential to have knowledge on high-risk profiles to guide personalized treatment.

The cross-species comparative genomic approach has been shown to be a powerful approach that may lead to key driver genes involved in tumor development, invasion, and progression. Indeed, several cancer driver genes and oncogenic pathways have been identified using this approach (Sweet-Cordero et al., 2005; Paoloni et al., 2009; Colak et al., 2010; Chen et al., 2013; Colak et al., 2013; Jin et al., 2019). In this study, we performed integrated transcriptomic and network analyses using several independent genome-wide gene expression profiling of human eHCCs and a rat model that we previously developed (Colak et al., 2010). Our aim is to identify a gene signature that is conserved across species and also expressed in blood and that may be involved in development of earliest phase of the disease and disease progression. We employed independent datasets of PBMC gene expression profiling for eHCC samples as well as eHCC dataset gathered from TCGA to estimate the diagnostic and prognostic significance of the discovered gene signature. In addition, functional network and pathway analyses were performed to identify significantly altered pathways that may be critical for early HCC transformation. Multivariate Cox regression analysis demonstrated that the identified gene signature predicted the disease outcome independent of other clinical variables. Our results may provide our gene signature’s potential to detect the disease in early stages by utilizing patients’ biological fluids, rather than using invasive procedures and prognostic significance for differentiating the high-risk patient group with a poor disease outcome from the low-risk group with a more favorable outcome.

## Materials and Methods

### Gene Expression Analysis

**FIGURE 1:**
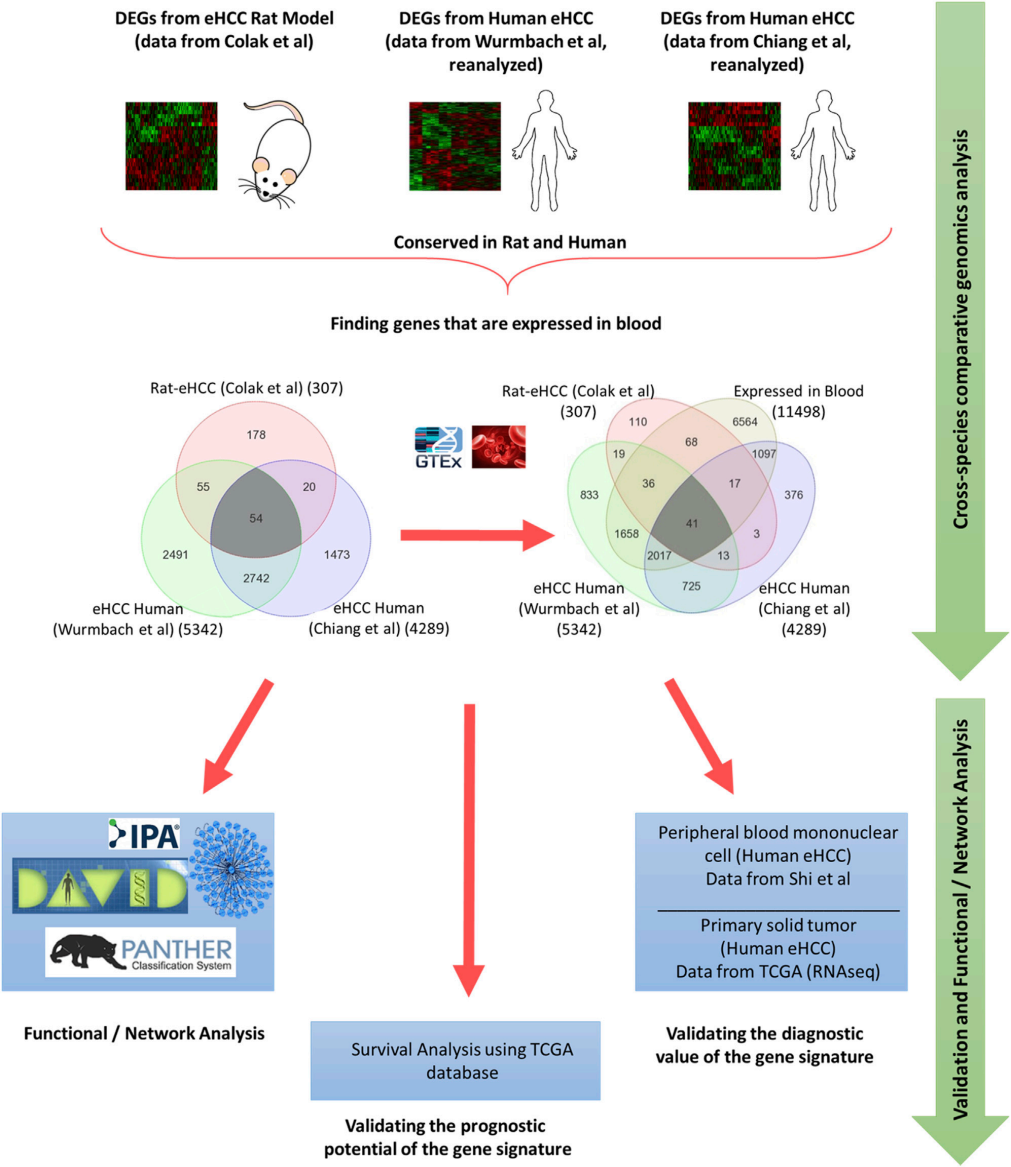
Schematic flowchart illustrating the methodology.

Integrated whole-genome gene expression analysis is performed using a rat eHCC dataset as well as several other publically available genomic datasets for human eHCC from independent studies, such as Chiang et al. (GSE9843) (Chiang et al., 2008) (n = 65) and Wurmbach et al. (GSE6764) (Wurmbach et al., 2007) (n = 28). The rat eHCC dataset is from our previous study (Colak et al., 2010), which consisted of 22 samples (6 eHCC, 8 regenerated liver, and 8 normal samples) that were probed using Applied Biosystems Rat Genome Survey microarray. Chiang *et al.‘s* dataset contained 91 HCV-related HCC tumor samples, of which 65 of them were eHCC that we used in our analysis. Wurmbach *et al.‘s* dataset included 75 samples, 28 of which we used (18 eHCC and 10 normal samples) in this study, hence included only data from patients with eHCC. All samples were probed using Affymetrix HGU133 Plus 2.0 array. We performed ANOVA to identify significant differentially expressed genes (DEGs) in each dataset. We adjusted the *p* values for multiple comparisons by false discovery rate (FDR) according to Benjamini-Hochberg step-up procedure (Benjamini and Hochberg, 1995). Significantly DEGs were defined as those with adjusted *p*-value < 0.05, and absolute fold change >1.5. The Venn diagram approach was used to find significant genes that are conserved in both rat and human HCC datasets. The GTEx portal (https://gtexportal.org/home/) is used to identify genes that are expressed in blood. An illustration of our methodology is shown in Figure 1.

### Functional Pathway, Gene Ontology Enrichment and Network Analyses

Functional pathway, gene ontology (GO) enrichment, and gene interaction network analyses were performed using (QIAGEN Inc., https://www.qiagenbioinformatics.com/products/ingenuity-pathway-analysis), (DAVID) (Dennis et al., 2003), Protein Analysis Through Evolutionary Relationships (PANTHER™) classification systems (Mi et al., 2017), and Network Analyst. (Xia et al., 2014; Xia et al., 2015). The DEGs were mapped to its corresponding gene object in the Ingenuity pathway knowledge base and gene interaction networks. A right-tailed Fisher’s exact test was used to calculate a *p*-value determining the probability that the biological function (or pathway) assigned to the data set is explained by chance alone (Colak et al., 2020).

### Independent Gene Expression Datasets for Validation

In order to validate the diagnostic value of our gene signature, we used two independently performed microarray and RNAseq datasets for early human HCCs. The first dataset was a PBMC microarray (Affymetrix Human Genome U133 U Plus 2.0 array) from Shi *et al’*s study (10 eHCCs and 10 normal controls) (Shi et al., 2014). The second dataset was RNAseq primary solid tumors from The *Cancer* Genome Atlas (TCGA) database that contained data from 421 samples (371 HCCs and 50 normal controls), including cases with HBV-infected, HCV-infected, and no virus infected HCCs. Among those 371 HCC samples, 171 were diagnosed as early HCC with stage 1 (TCGA-eHCC), which we also used for validation. We performed unsupervised PCA and two-dimensional hierarchical clustering by Pearson correlation with average linkage clustering using PARTEK Genomics Suite (Partek Inc., St. Louis, MO, United States). Moreover, we obtained an independent microarray gene expression profiling dataset from Gene Expression Omnibus database (GSE25097) (Ivanovska et al., 2011; Lamb et al., 2011) to validate the classifier built using our identified gene signature. The dataset includes 511 samples (268 hepatitis B virus (HBV) related HCC tumors and 243 adjacent normals). In order to identify the association of our gene signature with HCC tumor growth rate, we retrieved and reanalyzed a whole genome microarray expression dataset (GSE54236) (Villa et al., 2016). It contains 161 samples of HCC (n = 81) and normal (n = 80) tissue. Samples were probed using Agilent Whole Human Genome Oligo Microarrays. Tumor samples were divided into fast-growing (n = 20) and slow-growing tumors (n = 61) according to tumor doubling time, where the first quartile is considered the fast-growing tumors and the other three quartiles are the slow-growing tumors.

### Multivariate and Survival Analyses

Univariate and multivariate Cox regression analyses were used to investigate the prognostic value of our gene signature along with other clinical variables. A prognosis risk score for each patient was calculated (Aguirre-Gamboa et al., 2013), which is a linear combination of expression levels of our genes multiplied by a regression coefficient (*β*) of each gene extracted from the multivariate Cox proportional hazards regression model, using the following formula: prognosis risk score = expression of gene_1_ × β_1_+ expression of gene_2_ × *β*_2_ + … expression of gene_n_ × *β*_n_. We used an independent liver cancer dataset with detailed clinical data from TCGA RNAseq dataset that consisted of samples from 361 liver cancer patients. We used the median as a cutoff value for classifying patients into high and low risk groups.

Overall and recurrence free survival analyses were also performed. Survival curves were plotted using the Kaplan-Meier method, and significance between survival curves was calculated by the log-rank test. The Cox proportional-hazards regression for survival data was used to calculate hazard ratios. A *p*-value < 0.05 was considered statistically significant.

### HCC Classifier Model and Performance Evaluation

We used different machine learning algorithms to develop a predictive model for HCC using the 41 geneset. Several classifiers were built using K-Nearest Neighbor (KNN), Linear Support Vector Machine (SVM), Linear Discriminant Analysis (LDA) and Naïve Bayes (NB) to achieve the optimal classifier. The standardized gene expression values of our gene set were used as feature values. We estimated the classification performance on TCGA with 10-fold cross validation as well as training with TCGA (early stage HCC samples only, n = 221) and testing on an independent dataset (GSE25097) (n = 511 samples) (Ivanovska et al., 2011; Lamb et al., 2011). Hence, we tested the classifer performance on datasets that were not used for signature identification to confirm if our gene set is able to distinguish patients from normal controls. Four statistics measures were used: accuracy, specificity, sensitivity, and area under curve (AUC) that are defined as:

$$\text{Accuracy}=\;\frac{TP+TN}{TP+FN+FP+TN}$$

$$\text{Sensitivity}=\;\frac{TP}{TP+FN}$$

$$\text{Specificity}=\;\frac{TN}{TN+FP}$$

The TP, TN, FP, FN indicate true positive, true negative, false positive, and false negative, respectively. The classification analyses were performed using PARTEK Genomics Suite (Partek Inc., St. Louis, MO, United States).

### Statistical Analysis

Statistical analyses were conducted using MATLAB software packages (Mathworks, Natick, MA, United States) and PARTEK Genomics Suite (Partek Inc., St. Lois, MO, United States). All statistical tests were two-sided and *p*-value < 0.05 was considered statistically significant.

## Results

### Identification of a Blood-Based Gene Signature for Early HCC (eHCC)

**FIGURE 2:**
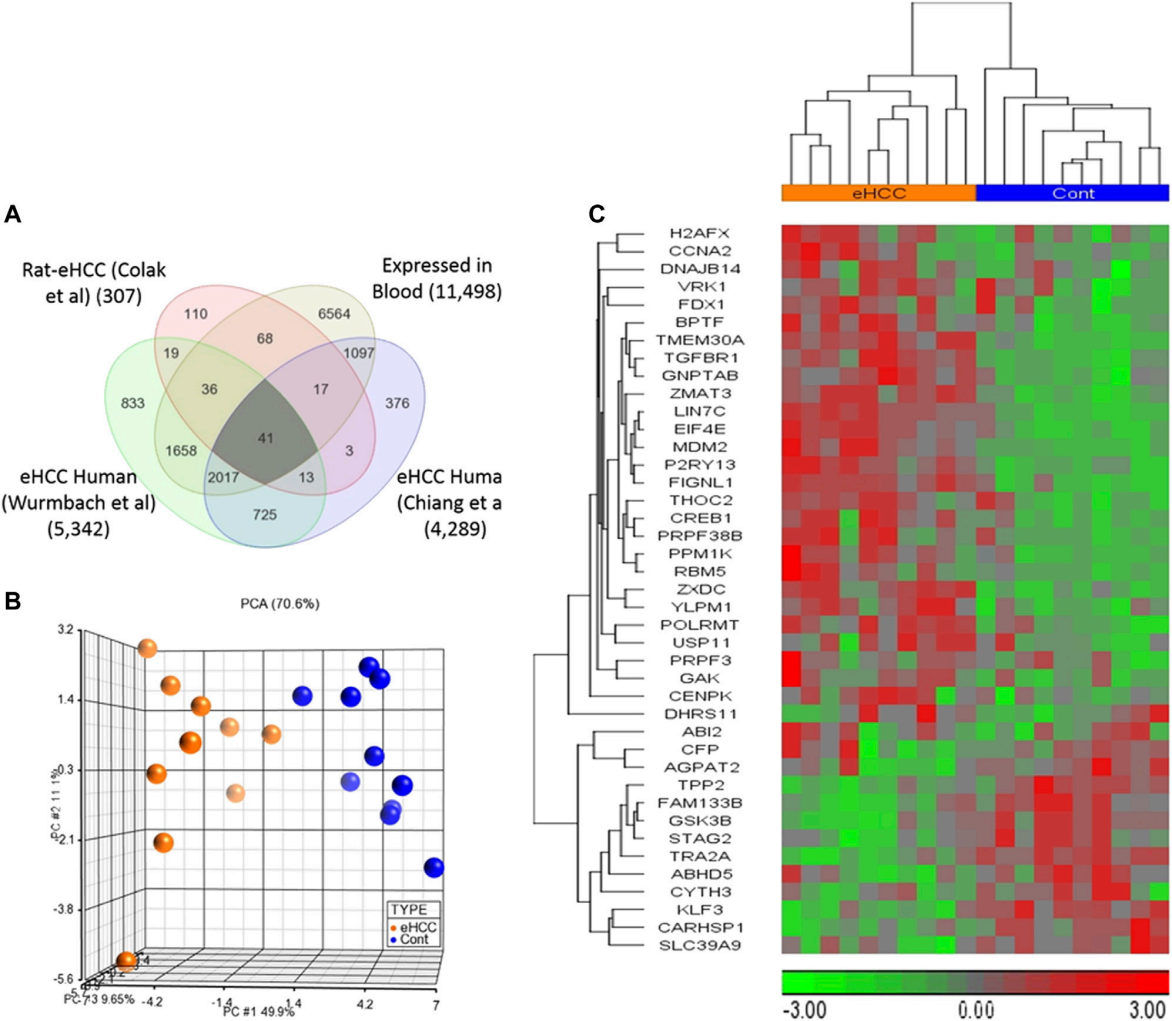
**(A)** Venn diagram demonstrates that there are 41 up- or down-regulated DEGs common among three different datasets and expressed in blood. **(B,C)** Unsupervised principal component analysis (PCA) and two-dimensional hierarchical clustering using our 41-geneset in microarray dataset derived from blood samples from patients with early HCC and controls (Shi et al., 2014). The PCA clearly separated eHCC patients from normal controls. The hierarchical clustering revealed two main sample-clusters: one with eHCC patients and the other with healthy controls. Samples are denoted in columns and genes are in rows. The orange color refers to eHCC samples, and blue for normal controls.

Genome-wide gene expression profiling provides valuable insight into the transcriptional changes that appear during the carcinogenic process beyond what may be obvious from studies evaluating only clinicopathologic characteristics. The investigation of human diseases using a combination of the human genome-wide molecular data and interactome may further provide an important viewpoint for understanding the molecular features of diseases (Al-Harazi et al., 2016; Al-Harazi et al., 2019). Here, we used several genome-wide gene expression profiling datasets from human eHCCs and a rat model of early HCC to conduct an integrative analysis. We identified 307 differentially expressed genes (DEGs) using our previous rat model of early HCC (Colak et al., 2010). We then analyzed human HCC data, focusing only on patients with early HCC, from Chiang et al. (Chiang et al., 2008) and Wurmbach et al. (Wurmbach et al., 2007), that revealed 4,289 and 5,342 DEGs that were significantly dysregulated in patients compared to normal controls (adjusted *p*-value < 0.05 and absolute fold-change > 1.5). The Venn diagram approach indicated 2,796 DEGs were shared by both human datasets and 54 of which were conserved across both species (Figure 1). Cross-species conserved genes in eHCC that are also expressed in blood revealed 41 genes that we define as “41-gene signature” (Figure 2A and Table 1). We then performed functional and network analyses as well as extensive validations for the diagnostic and prognostic potential of the identified geneset (Figure 1).

**TABLE 1:** The 41-gene signature identified in this study for early HCC.

Symbol	Gene Title
ABHD5	Abhydrolase domain containing 5
ABI2	abl-interactor 2
AGPAT2	1-acylglycerol-3-phosphate O-acyltransferase 2
BPTF	bromodomain PHD finger transcription factor
CARHSP1	calcium regulated heat stable protein 1, 24 kDa
CCNA2	cyclin A2
CENPK	centromere protein K
CFP	complement factor properdin
CREB1	cAMP responsive element binding protein 1
CYTH3	cytohesin 3
DHRS11	Dehydrogenase/reductase (SDR family) member 11
DNAJB14	DnaJ (Hsp40) homolog, subfamily B, member 14
EIF4E	eukaryotic translation initiation factor 4 E
FAM133B	family with sequence similarity 133, member B
FDX1	ferredoxin 1
FIGNL1	fidgetin-like 1
GAK	cyclin G associated kinase
GNPTAB	N-acetylglucosamine-1-phosphate transferase, alpha and beta subunits
GSK3B	glycogen synthase kinase 3 beta
H2AFX	H2A histone family, member X
KLF3	Kruppel-like factor 3 (basic)
LIN7C	lin-7 homolog C (C. elegans)
MDM2	MDM2 proto-oncogene, E3 ubiquitin protein ligase
P2RY13	purinergic receptor P2Y, G-protein coupled, 13
POLRMT	Polymerase (RNA) mitochondrial (DNA directed)
PPM1K	protein phosphatase, Mg2+/Mn2+ dependent, 1 K
PRPF3	pre-mRNA processing factor 3
PRPF38B	pre-mRNA processing factor 38 B
RBM5	RNA binding motif protein 5
SLC39A9	solute carrier family 39, member 9
STAG2	stromal antigen 2
TGFBR1	transforming growth factor, beta receptor 1
THOC2	THO complex 2
TMEM30A	transmembrane protein 30 A
TPP2	tripeptidyl peptidase II
TRA2A	transformer 2 alpha homolog (Drosophila)
USP11	ubiquitin specific peptidase 11
VRK1	vaccinia related kinase 1
YLPM1	YLP motif containing 1
ZMAT3	zinc finger, matrin-type 3
ZXDC	ZXD family zinc finger C

### Diagnostic and Prognostic Significance of the 41-Gene Signature

**FIGURE 3:**
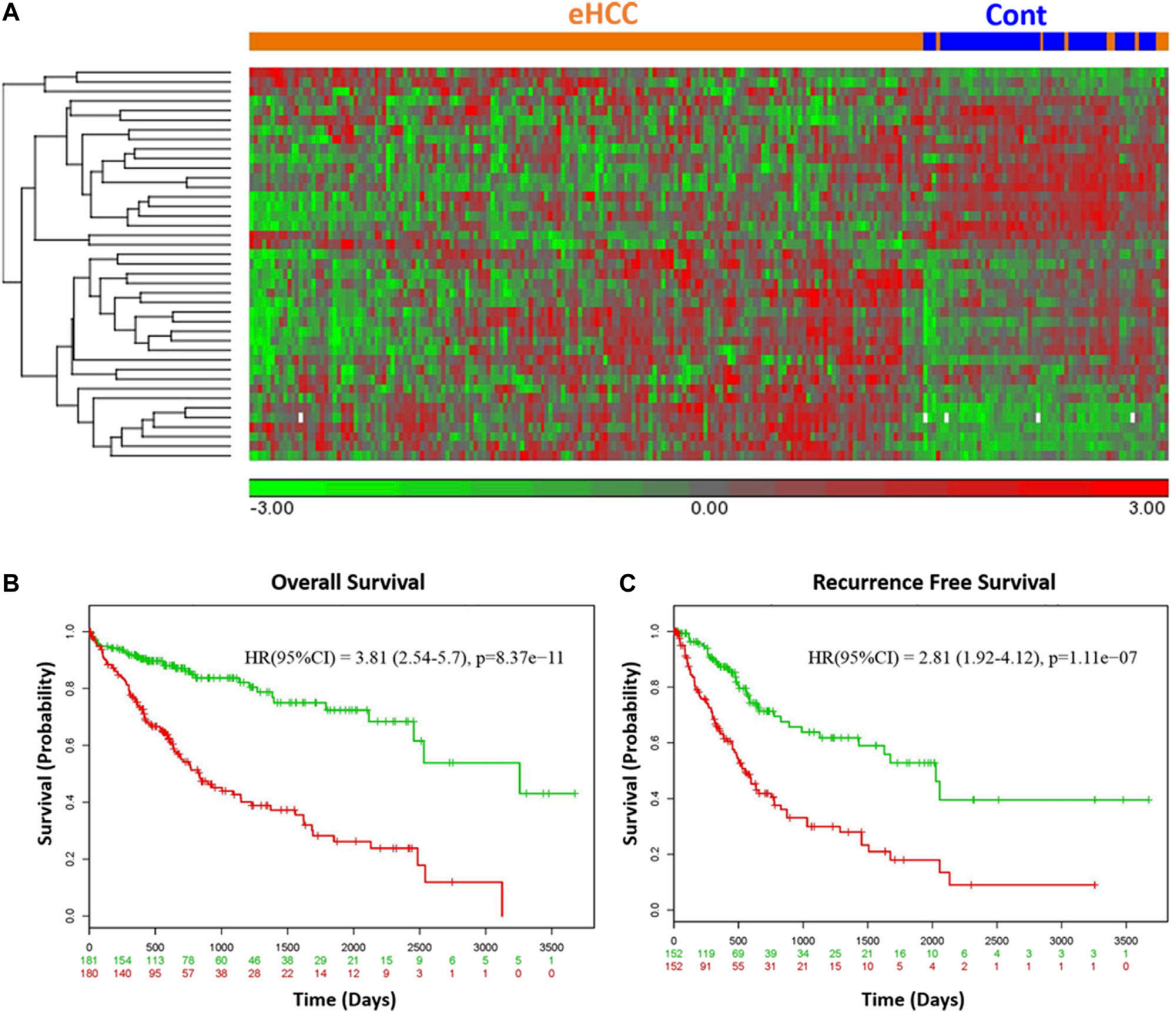
**(A)** Unsupervised two-dimensional hierarchical clustering of TCGA using our 41-gene signature. The hierarchical clustering distinguished samples as either eHCC or normal controls. Samples are indicated in columns, while genes are in rows. Orange indicates eHCC and blue control samples. The expression level of each gene across the samples is normalized to (−3, 3). Overall **(B)** and recurrence free survival **(C)** of 41-gene signature using TCGA dataset. Kaplan-Meier curves for risk groups, “+” mark indicates censoring samples. Horizontal axis denotes time to event. Red/Green curves indicate High/Low-risk groups respectively. The red and green numbers below horizontal axis represent the number of individuals not presenting the event of the corresponding risk group along time.

We employed two independent datasets for early human HCCs to confirm the diagnostic significance of the 41-geneset. The first dataset is a microarray data extracted from PBMC RNA samples of patients with HCC (Shi et al., 2014). The unsupervised principal components analyses (PCA) and two-dimensional hierarchical clustering using our 41-geneset clearly distinguished patients as either eHCC or normal controls in both datasets (Figures 2B,C, respectively). Moreover, we also tested the 41-gene using mRNAseq data from TCGA (n = 421). We selected only the patients with eHCC and controls (n = 171 patients, 50 controls). The unsupervised two-dimensional hierarchical clustering using the 41-geneset separated samples into two main clusters with differing gene expression patterns, one cluster with eHCC samples and the other one mainly composed of controls (Figure 3A).

We confirmed the prognostic significance of our gene signature using TCGA HCC cohort for overall as well as recurrence-free survival. A prognostic risk score is calculated using our 41-gene as described in the methods section and patients are classified as high or low risk using the median risk score as cutoff. The survival analyses demonstrated that high risk score cohort were significantly associated with poor disease outcome (Figures 3B,C). Indeed, patients in the high-risk group (n = 180) had a significantly poorer prognosis with hazard ratio (HR) = 3.81 (*p* = 8.37 × 10^−11^, 95% confidence interval [CI]: 2.54–5.7) for overall survival (Figure 3B) and HR = 2.81 (95% CI = 1.92–4.12; *p* = 1.1 × 10^-7^) for the recurrence-free survival (Figure 3C).

### The 41-Gene Signature Is an Independent Prognostic Factor

We used Cox’s regression models to determine whether 41-gene signature could prognosticate disease outcome independent of other clinical factors. Univariate regression analysis of the TCGA dataset showed that the 41-gene risk score (HR = 3.81, 95% CI = 2.54–5.70; P = 8.37 × 10^-11^), stage (HR = 2.56, 95% CI = 1.75–3.7; P = 1.38 × 10^-6^), and macrovascular invasion (HR = 2.20, 95% CI = 1.00–4.84; P = 0.05) were significantly associated with patient prognosis, while age, gender, grade and AFP showed no significant association with overall survival. However, the multivariate regression analysis revealed that only the 41-gene signature predicted the outcome of HCC independent of other clinical variables (HR = 3.24, 95% CI = 1.95–5.38; P = 5.58 × 10^-6^) (Table 2).

**TABLE 2:** Univariate and multivariate analyses associated with overall survival (OS).

Variables	Univariate analysis		Multivariate analysis	
	p value	HR (95% CI)	p value	HR (95% CI)
Age (years) < 50 vs ≥ 50	0.71	0.92 (0.57–1.46)	0.19	0.68 (0.39–1.20)
Gender Female vs Male	0.25	1.25 (0.85–1.84)	0.98	0.99 (0.63–1.56)
Stage III-IV vs I-II	**1.38e-06**	2.56 (1.75–3.7)	0.06	1.58 (0.98–2.63)
Grade G3-4 vs G1-2	0.41	1.18 (0.8–1.72)	1.35	1.07 (0.69–1.69)
AFP (ng/ml) < 400 vs ≥ 400	0.86	0.96 (0.59–1.57)	–	–
Macrovascular invasion vs None	**0.05**	2.20 (1.00–4.84)	0.37	1.47 (0.63–3.42)
Microvascular invasion vs None	0.49	1.18 (0.75–1.85)	0.75	1.08 (0.66–1.78)
Risk score High vs Low	**8.37e-11**	3.81 (2.54–5.70)	**5.58e-06**	3.24 (1.95–5.38)

Bold indicates significance. **Abbreviations:** CI, confidence interval; HR, hazard ratio; AFP, serum alpha-fetoprotein.

### Association of the 41-Gene Signature With the HCC Dynamic Progression

**FIGURE 4:**
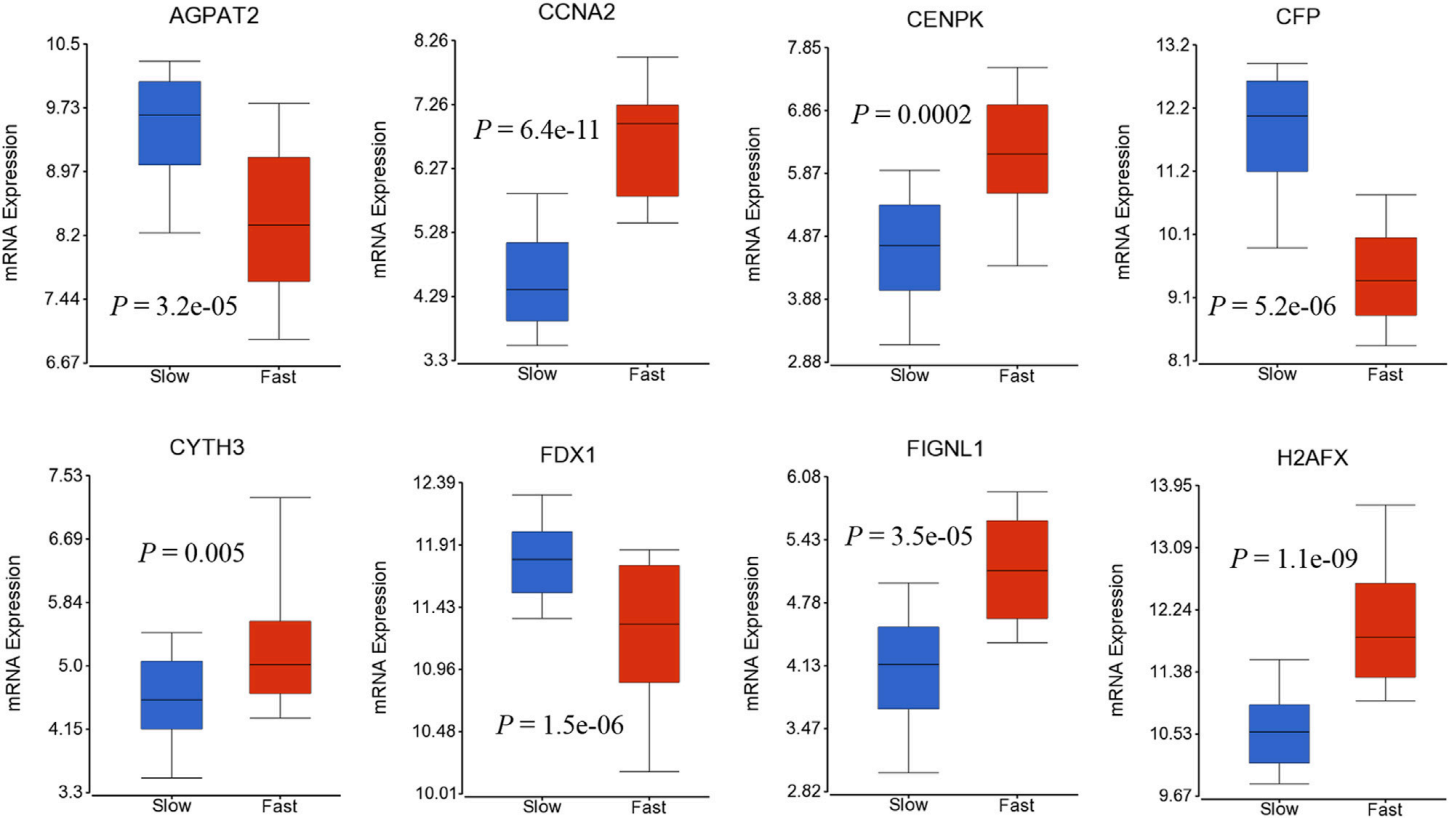
Boxplots displaying the gene expression levels of selected genes in fast-growing (≤53 days; *n* = 19) and slow-growing (>54 days; *n* = 59) using dataset by (Villa et al., 2016).

We also investigated if genes in 41-geneset are involved in dynamic progression of the disease using a transcriptomic dataset that included information on HCC patients at presentation according to fast- or slow-growth speed (Villa et al., 2016).The tumor doubling times ranged from 30 to 621 days were divided into quartiles and classified as fast-growing (≤53 days; n = 19) and slow-growing (>54 days; n = 59) (Villa et al., 2016). Testing the mRNA expression of genes in 41-geneset revealed that *CCNA2*, *CENPK*, *CYTH3*, *FIGNL1*, and *H2AFX* have significantly higher level of expression in fast-growing tumors compared to slow-growing tumors and *AGPAT2, CFP*, and *FDX1* have significantly lower level of expression in fast-growing tumors compared to slow-growing tumors (Figure 4).

### Gene Ontology Enrichment, Pathway and Network Analyses

**FIGURE 5:**
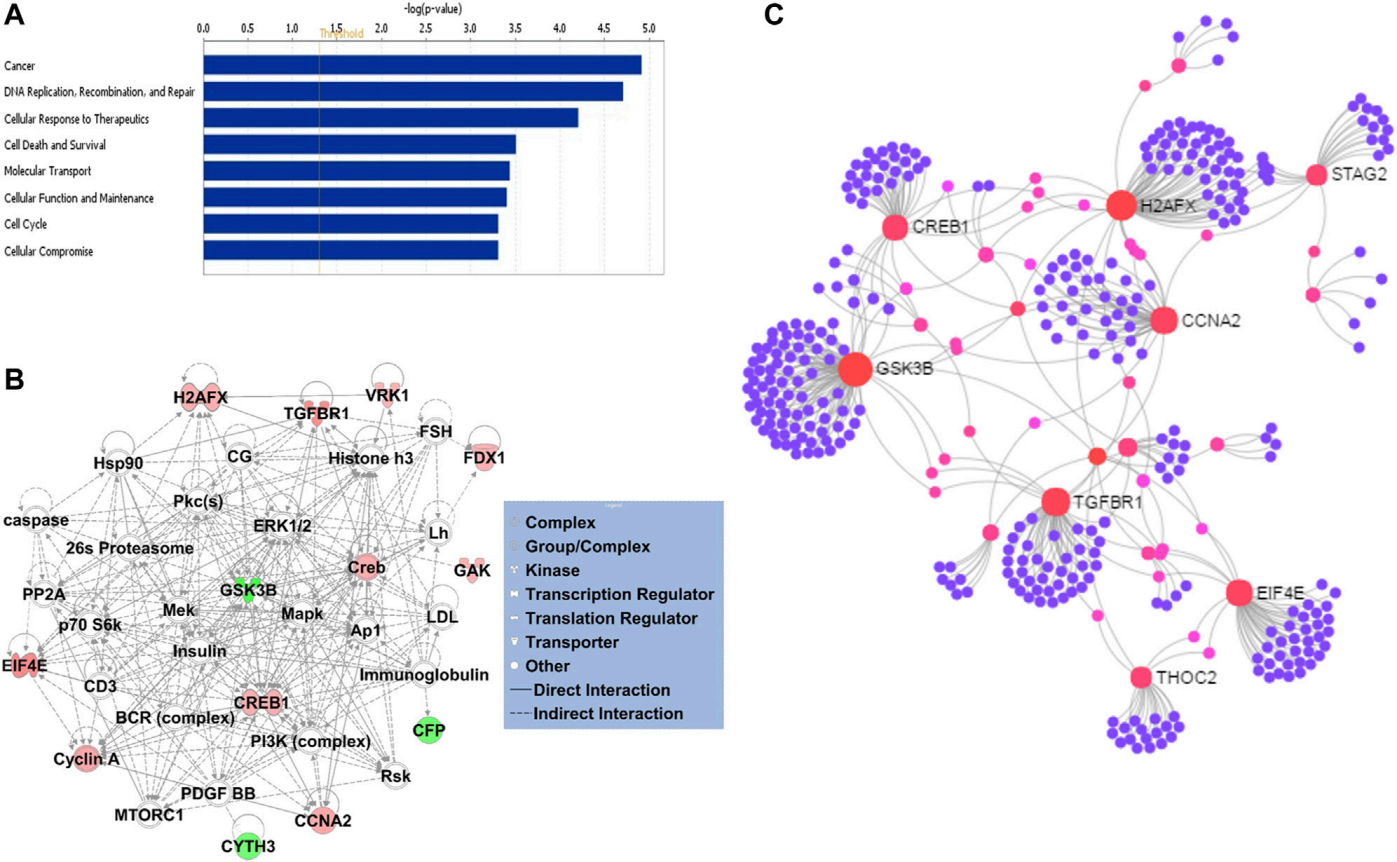
**(A)** The GO and functional, and **(B)** gene interaction network analyses of the gene signature. Red/green indicates higher/lower expression in eHCCs compared to controls. Straight lines are for direct interactions and dashed lines for indirect ones. **(C)** A subnetwork of the 41-gene signature with the hub genes labelled. The size of nodes is proportional to their betweenness centrality values.

Gene ontology enrichment and functional analyses of the blood-based gene signature revealed that 41-gene signature is enriched for functional categories related to DNA replication, recombination, and repair, RNA metabolic process, cell cycle, tissue development, cell death, and survival (Figure 5A; Table 3). The significantly altered canonical pathways include a number of cancer pathways, including Cell Cycle: G1/S Checkpoint Regulation, ATM Signaling, Antiproliferative Role of TOB in T Cell Signaling, PI3K/AKT Signaling, Wnt/β-catenin and Wnt/Ca^2+^ pathways. The gene interaction network of 41-gene is displayed in Figure 5B. Furthermore, network analysis indicated hub genes that may be potentially important in eHCC transformation, including *EIF4E, GSK3β, CCNA2, H2AFX, TGFBR1* and *CREB1* (Figure 5C).

**TABLE 3:** The enriched GO Biological Processes associated with the identified 41- gene signature.

GO Term	%*	p-value	Selected Genes
Cell cycle	24	1.13E-02	VRK1, EIF4E, TGFBR1, GSK3B, MDM2, H2AFX, CENPK, CCNA2, STAG2, GAK
Regulation of cell development	17	1.19E-02	EIF4E, TGFBR1, GSK3B, CREB1, MDM2, TMEM30A, GAK
RNA splicing	12	1.27E-02	TRA2A, RBM5, PRPF3, THOC2, PRPF38B
Response to growth factor	15	1.40E-02	BPTF, TGFBR1, GSK3B, CREB1, MDM2, CCNA2
Macromolecular complex subunit organization	29	1.85E-02	VRK1, BPTF, TGFBR1, GSK3B, CREB1, RBM5, ABI2, MDM2, H2AFX, PRPF3, CENPK, GAK
Neuron differentiation	20	1.94E-02	EIF4E, TGFBR1, GSK3B, CREB1, MDM2, THOC2, TMEM30A, GAK
RNA metabolic process	44	1.95E-02	CARHSP1, TGFBR1, CREB1, YLPM1, TRA2A, ZXDC, RBM5, PRPF3, POLRMT, EIF4E, BPTF, GSK3B, MDM2, H2AFX, THOC2, CCNA2, PRPF38B, KLF3
Viral process	17	2.11E-02	EIF4E, CREB1, ABI2, MDM2, H2AFX, THOC2, CCNA2
Multi-organism cellular process	17	2.18E-02	EIF4E, CREB1, ABI2, MDM2, H2AFX, THOC2, CCNA2
Gene expression	46	3.29E-02	CARHSP1, TGFBR1, CREB1, YLPM1, TRA2A, ZXDC, RBM5, PRPF3, CFP, POLRMT, EIF4E, BPTF, GSK3B, MDM2, H2AFX, THOC2, CCNA2, PRPF38B, KLF3

*Indicates the percentage of genes among the identified blood-based gene signature that are involved in each GO biological process term.

### Classification Model for HCC and Validation Analysis

We used several classification algorithms, including KNN, SVM, LDA, and NB, to design an optimal classifier for HCC with the 41-gene signature. The KNN (k = 1 and Pearson’s Dissimilarity distance measure) has outperformed the others and displayed the best classification power. We developed multiple KNN classifiers using different parameters and then tested their performance on independent validation datasets (Table 4). First, we used the Chiang et al. dataset as training and the TCGA dataset (consisting of only early stage HCC cases and controls; the TCGA-eHCC dataset) as testing. The clasisifcation model achieved a high prediction accuracy of 95% and sensitivity, specificity and AUC of 95, 96 and 95%, respectively. We then used the TCGA dataset that included all HCC samples with all disease stages (n = 421) as well as using only early stage HCC samples (n = 221) as a training dataset to develop the KNN classifier and performed 10-fold cross-validations. The 41-gene classifier displayed high prediction accuracy of 95 and 93% using all HCC samples and early HCC samples, respectively, and both displayed much superior performance compared to using serum biomarker AFP (Table 4). Finally, we trained the classifier on early stage HCC samples from TCGA (n = 221) and tested the classifier performance on an independent validation dataset (GSE25097) that included 511 samples (268 HBV-infected HCC tumors and 243 normals). Again, the designed 41-gene model has achieved a superior classification performance (accuracy of 90%) compared to using AFP (accuracy = 53%) alone (Table 4).

**TABLE 4:** Classification analyses. Prediction performance of KNN classification on different validation datasets.

	Accuracy	Sensitivity	Specificity	AUC
*Chiang et al as training (n = 65) and TCGA (Stage 1) dataset for validation (n = 221)*				
41 genes	0.95	0.95	0.96	0.95
AFP	0.75	0.90	0.16	0.54
41 genes + AFP	0.95	0.94	0.96	0.95
*TCGA with 10-fold cross validation (n = 421)*				
41 genes	0.95	0.95	0.98	0.96
AFP	0.82	0.90	0.26	0.58
41 genes + AFP	0.95	0.95	0.98	0.97
*TCGA (Stage 1) with 10-fold cross validation (n = 221)*				
41 genes	0.93	0.92	0.94	0.93
AFP	0.71	0.81	0.38	0.60
41 genes + AFP	0.93	0.94	0.92	0.93
*TCGA (Stage 1) as training (n = 221) and GSE25097 for validation (n = 511)*				
41 genes	0.90	0.97	0.83	0.90
AFP	0.53	0.72	0.34	0.53
41 genes + AFP	0.90	0.98	0.82	0.90

*Abbreviations: AUC, area under curve; TCGA, The Cancer Genome Atlas; AFP, serum alpha-fetoprotein.

## Discussion

Hepatocellular carcinoma (HCC) is the fourth leading global cause of cancer-related death in the world (Bray et al., 2018). It is a malevolent disease that develops furtively and is frequently diagnosed at advanced stages, resulting in a poor prognosis. Robust early detection biomarkers are needed to detect the disease at its onset as well as to enable prognostic estimation to improve the outcomes of the patients with HCC (Colak et al., 2010; Shi et al., 2014; Allain et al., 2016; Wang et al., 2017; Guan et al., 2020; Li et al., 2020). Currently there are a number of biomarkers that have been used as HCC diagnostic markers, such as AFP that has been commonly used for its feasibility and low cost. Other biomarkers include AFP-L3, des-gamma-carboxyprothrombin (DCP), glypican-3, insulin-like growth factor (IGF)-1, and hepatocyte growth factor (HGF) (Schütte et al., 2014). However, alpha-Fetoprotein bears low specificity owing to its presence in other cancer types (Schütte et al., 2014). Recent genomic works focus on using RNA-related approaches such as mRNA (Wang et al., 2017; Guan et al., 2020; Zhou et al., 2020) and miRNA signatures (Liu et al., 2017; Bai et al., 2018a), long non-coding RNAs (Arun et al., 2018; Ma et al., 2020) targeted mRNAs for critical genes (Lin et al., 2016), and DNA based approaches such as driver mutations or circulating tumor DNAs (Yang et al., 2019b; Cai et al., 2019). However, some of the highly predictive biomarkers suffer from requiring invasive procedures or not able to detect the early HCC development. Therefore, there is clear and unmet need for biomarkers that are more informative and sensitive with high accuracy for the early detection and prognostication of HCC. In this study, we performed an integrated transcriptomic and network-based analyses of eHCC using several whole-genome gene expression profiling datasets from human eHCCs and rat model of eHCC that we developed previously (Colak et al., 2010). We aimed to identify a gene signature that is conserved across species that detect the disease in early stages in patients’ biological fluids instead of using invasive techniques and also have prognostic significance for differentiating the high-risk patient group from the low-risk group.

We identified 41-gene signature and validated its diagnostic and prognostic potential using independent datasets from large cohort of HCC patients (over 600 cases), including gene expression profiling of PBMC from patients with eHCC as well as the eHCC cohort in TCGA. As we used comparative genomic analysis of rat and human eHCCs, the resulting gene signature had conserved genomic profile of the tumor. The previous studies have demonstrated that cross-species comparative genomic method is a robust methodology for identifying essential genes that are involved in tumorigenesis, invasion and progression, and hence has therapeutic promise (Sweet-Cordero et al., 2005; Paoloni et al., 2009; Colak et al., 2010; Colak et al., 2013). Several driver mutations and critical pathways in disease progression have been identified using this approach (Sweet-Cordero et al., 2005; Jin et al., 2019).

Our results reveal alterations in several cancer-related pathways as well as key hub genes, including *EIF4E, GSK3β, CCNA2, H2AFX, TGFBR1, CREB1, TGFBR1, THOC2, ZMAT3,* and *STAG2,* potentially critical in early HCC transformation and progression*.* The *EIF4E* aids in translation initiation by uniting the 4 F complex. *EIF4E* behaves as a proto-oncogene involved in transformation and tumorigenesis. *EIF4E* have previously been linked to human cancer. It has been reported that *EIF4E* protein contributes to malignant transformation and progression by enhancing translation of cancer-related mRNAs in eukaryotic cells (Jiang et al., 2016). In addition, there are several studies investigated the association of *EIF4E* and human tumors such as lung cancer (Seki et al., 2010), colorectal cancer (Niu et al., 2014; Diab-Assaf et al., 2015; Slattery et al., 2017), breast cancer (Heikkinen et al., 2013; Hu et al., 2014), and head and neck carcinoma (Nathan et al., 1997; Culjkovic and Borden, 2009). In patients with HCC, the high expression of *EIF4E* is associated to tumorigenesis (Jiang et al., 2016). Moreover, patients with increased *EIF4E* expression had a poorer prognosis compared to patients with decreased *EIF4E* expression (Jiang et al., 2016). Moreover, it is also found in another study that patients with high expression levels of *EIF4E* had more recurrent liver metastasis (Li et al., 2016). However, the molecular role of *EIF4E* in eHCC blood samples has not been well-defined. Herein, we found that *EIF4E* is one of the significant genes that show high expression in blood samples from eHCC compared to normal samples (Figure 2C and Supp Figure 1). The functional analysis indicates that *EIF4E* is associated with several GO biological functions including the cell cycle, regulation of cell development, RNA metabolic processes, viral processes, and gene expression (Table 2).

Our findings indicated that the expression of *GSK3β* is significantly lower in eHCC tumor samples in comparison to normal samples in blood (Figure 2C and Supp Figure 1). Previous studies indicated that the GSK-3 gene family plays a significant role in various human cancers, including hepatocarcinoma (McCubrey et al., 2014; Mancinelli et al., 2017). The authors have also reported that GSK-3 has an effect on tumor progression by stabilizing the beta-catenin complex components (McCubrey et al., 2014; Mancinelli et al., 2017). Furthermore, it has been reported that the dysregulation of *GSK3β* phosphorylation and inhibition of *GSK3β* activity contributes to hepatocarcinogenesis (Desbois-Mouthon et al., 2002; McCubrey et al., 2014). Previous studies indicate that *GSK3β* is possibly a suppressor gene in HCC tumors, due to the loss of *GSK3β* expression and/or activity participating in HCC progression (McCubrey et al., 2014; Cervello et al., 2017; Fang et al., 2019).

Our results also revealed that *CCNA2* and *H2AFX* have significantly higher level of mRNA expression in both tissue and blood samples from eHCC (Figure 2C, 3A, and Supp Figure 1). *CCNA2* is considered a biomarker for ER-positive breast cancer prognosis and it can help monitor tamoxifen efficacy (Gao et al., 2014). In some recent studies, *CCNA2* was suggested to be a prognostic biomarker for liver carcinoma, as it may help in developing an effective therapeutic and/or preventative approach for HCC (Bai et al., 2018b; Zhang et al., 2018; Wu et al., 2019). The *H2AFX* and its phosphorylated C-terminal (*γ*-H2AX) are potential regulators of DNA repair and are essential in DNA damage response (Celeste et al., 2003; Bassing and Alt, 2004; Fernandez-Capetillo et al., 2004). Matsuda *et al.* reported that histological grades of HCC are associated with the level of labeling index (LI) of *γ*-H2AX, which indicates that *γ*-H2AX may play a significant role in the development of HCC, particularly throughout the early stages of carcinogenesis (Matsuda et al., 2013). Interestingly, we also found that *CCNA2* and *H2AFX* have significantly higher level of expression in fast-growing tumors compared to slow-growing tumors (Figure 4), indicating their association with rapid tumor growth.

Gene ontology, gene network and pathway analyses of the 41-gene signature revealed enrichment of biological functions including DNA replication, recombination and repair, cellular response to therapeutics, cell cycle, cell death and survival, and regulation of cell development (Figure 5A). DNA repair genes are overexpressed in cancer tissues, and hence develop larger DNA repair capacity compared to normal tissues (Kirkali et al., 2011; Lin et al., 2016). Consequently, numerous DNA damage signals and DNA repair pathways may have a major influence on prognosis and response to therapy for different types of cancers (Dizdaroglu, 2015; Lin et al., 2016). The cell death process appears in almost all types of human liver diseases including HCC, and it is considered a sensitive parameter for the detection of disease (Luedde et al., 2014). Our results revealed significantly dysregulated genes that are associated with cell death and survival, including *TGFBR1, RBM5, THOC2, USP11, MDM2, TPP2, EIF4E, VRK1, CCNA2, ZMAT3, AGPAT2, H2AFX, CREB1, FIGNL1,* and *GSK3B*. Identifying the characteristics of these genes and their network of interaction is important to understand the pathophysiology of HCC and discover new therapeutic targets for the disease.

## Conclusion

In summary, our study reveals several genes and pathways that are essential for eHCC transformation and validate our gene signature’s potential to detect the disease in patients’ biological fluids instead of utilizing invasive techniques and predict the disease prognosis. Having genomic biomarkers with diagnostic and prognostic potential is invaluable for HCC patients for early detection of the disease at its earliest stage as well as differentiating the high risk patient group with poor disease outcome from the low risk ones. Our results suggest that the integrated cross-species transcriptomic analysis with the gene networks may provide a robust methodology for understanding the key biological programs in eHCC and may lead to better diagnosis, prognosis and therapeutic choices.

## Data Availability

Publicly available datasets were analyzed in this study. These data can be found here: The Cancer Genome Atlas (TCGA) and the NCBI Gene Expression Omnibus.

## Supplementary Material

Supplementary Figure 1 Boxplots display the gene expression levels of selected genes in blood of early HCC and controls using dataset from (Shi et al., 2014).

## Abbreviations

AFP, alpha-fetoprotein; DCP, des-gamma-carboxyprothrombin; DEG, differentially expressed gene; eHCC, early HCC; FDR, false discovery rate; GO, gene ontology; GTEx, Genotype-Tissue Expression; HCC, hepatocellular carcinoma; HGF, hepatocyte growth factor; IGF, insulin-like growth factor; OS, Overall Survival; PBMC, peripheral blood mononuclear cell; PCA, principal component analysis; PPI, protein-protein interaction; TCGA, The Cancer Genome Atlas.
