# Supplementary figures and images for: Identification of Gene Signature as Diagnostic and Prognostic Blood Biomarker for Early Hepatocellular Carcinoma Using Integrated Cross-Species Transcriptomic and Network Analyses
Source: Front Genet. 2021 Sep 29;12:710049. doi: 10.3389/fgene.2021.710049 (PMC8511318; doi:10.3389/fgene.2021.710049)

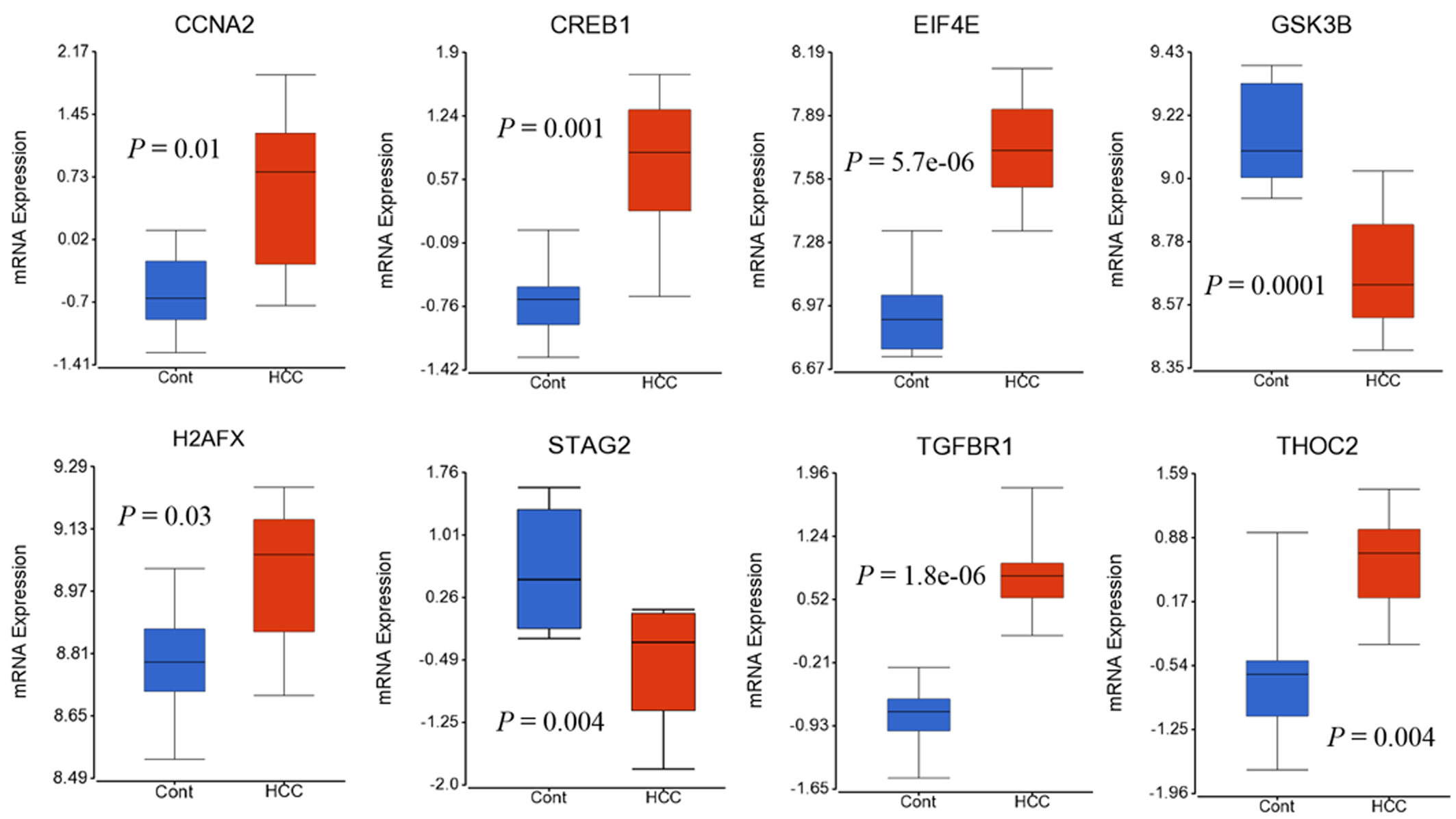

Supplement: Supplementary file 1 [file Image1.TIF]
